# Regulation and Maturation of the *Shewanella oneidensis* Sulfite Reductase SirA

**DOI:** 10.1038/s41598-020-57587-6

**Published:** 2020-01-22

**Authors:** Kenneth L. Brockman, Sheetal Shirodkar, Trevor J. Croft, Rini Banerjee, Daad A. Saffarini

**Affiliations:** 1 Department of Biological Sciences, University of Wisconsin Milwaukee, Milwaukee, Wisconsin USA; 2 Present Address: Department of Microbiology & Immunology, Medical College of Wisconsin, Milwaukee, Wisconsin USA; 3 Present Address: Amity University Uttar Pradesh, Amity Institute of Biotechnology, Noida, India; 4 Present Address: Department of Microbiology and Molecular Genetics, University of California Davis, Davis, California USA

**Keywords:** Bacteriology, Element cycles, Water microbiology

## Abstract

*Shewanella oneidensis*, a metal reducer and facultative anaerobe, expresses a large number of *c*-type cytochromes, many of which function as anaerobic reductases. All of these proteins contain the typical heme-binding motif CXXCH and require the Ccm proteins for maturation. Two *c*-type cytochrome reductases also possess atypical heme-binding sites, the NrfA nitrite reductase (CXXCK) and the SirA sulfite reductase (CX_12_NKGCH). *S. oneidensis* MR-1 encodes two cytochrome *c* synthetases (CcmF and SirE) and two apocytochrome *c* chaperones (CcmI and SirG). SirE located in the *sir* gene cluster is required for the maturation of SirA, but not NrfA. Here we show that maturation of SirA requires the combined function of the two apocytochrome *c* chaperones CcmI and SirG. Loss of either protein resulted in decreased sulfite reductase. Furthermore, SirA was not detected in a mutant that lacked both chaperones, perhaps due to misfolding or instability. These results suggest that CcmI interacts with SirEFG during SirA maturation, and with CcmF during maturation of NrfA. Additionally, we show that CRP regulates expression of *sirA* via the newly identified transcriptional regulatory protein, SirR.

## Introduction

*Shewanella* species are facultative anaerobes that are abundant in freshwater and marine environments and are best known for their ability to use metals as electron acceptors for anaerobic respiration (see Fredrickson *et al*. for review^1^). *S. oneidensis* MR-1 is one of the best studied members of the *Shewanella* genus and the most diverse with regard to the electron acceptors it uses for respiration. These include O_2_, fumarate, NO_3_^−^, NO_2_^−^, trimethylamine N-oxide (TMAO), dimethylsulfoxide (DMSO), metal oxides, thiosulfate, and sulfite^2–10^. In addition, *S. oneidensis* can reduce radionuclides and toxic metals such as Tc, U, and Cr^11–15^. The ability of *S. oneidensis* to use this wide range of electron acceptors is partly attributed to the large number of *c*-type cytochromes encoded by its genome^16,17^.

Dissimilatory sulfite reduction is a key intermediate step in sulfate reduction and has been extensively studied in many sulfate reducing prokaryotes such as *Desulfovibrio vulgaris* and *Archeoglobus fulgidus* (for review see^18^) and in *Salmonella typhimurium*. *Desulfovibrio*, *Archeoglobus* and *Salmonella* species all utilize a siroheme-containing enzyme to reduce sulfite to sulfide^19–21^. In contrast, the sulfite reductase of *Shewanella oneidensis* MR-1 lacks siroheme. In *S. oneidensis*, the terminal sulfite reductase consists of SirA, an octaheme *c* cytochrome, SirC, an Fe-S protein, and SirD, which is predicted to be a membrane-bound quinol oxidase, based on similarity to the quinol oxidase component of the formate-dependent nitrite reductases^10^. A similar system involved in sulfite reduction has been identified in *Wolinella succinogenes*^22^.

c-type cytochromes are characterized by the covalent attachment of heme b vinyl groups to the two cysteines present in the typical CXXCH heme-binding motif^23^. In α and γ Proteobacteria, the System I cytochrome maturation proteins, CcmABCDEFGH, attach a heme moiety to the CXXCH motif (see^23–25^ for review). The final step in heme attachment and enzyme maturation requires transfer and covalent linkage of heme by the heme lyase complex. In Escherichia coli, CcmFH form the complex that functions as a heme lyase, whereas in other Gram-negative bacteria such as *Rhodobacter capsulatus*, the heme lyase complex consists of CcmFHI^26^. Similar to other Gram-negative bacteria, maturation of c-type cytochromes in *S. oneidensis* requires proteins encoded by genes in the *ccmABCDE* and *ccmFGH* operons. Mutations in the *ccm* genes lead to complete loss of mature c-type cytochromes^27,28^. SO_0265 was identified as *ccmI*, and was initially determined to have a nonessential role in cytochrome c maturation in *S. oneidensis*^28^, but subsequently found to have a role in maturation of canonical heme binding motifs and to be required for maturation of the nitrite reductase, NrfA^29^. However, it remains unknown if or what role CcmI plays in maturation of the sulfite reductase.

In addition to heme attachment to the typical CXXCH motif, specialized heme lyase systems can attach heme b to atypical sites, such as CXXCK and CX_15_CH^30,31^. In *E. coli*, ligation of heme to the CXXCK site of NrfA requires the heme lyase NrfEFG^30^. In *Wolinella succinogenes*, two distinct heme lyases, NrfI and CcsA1, are required for heme attachment to the CXXCK binding sites of the nitrite reductase and the CX_15_CH binding site of the sulfite reductase, respectively^32,33^. Two atypical heme-binding sites have been identified in *S. oneidensis* c-type cytochromes. The CXXCK motif is found in the periplasmic nitrite reductase NrfA^34^, and the CX_12_NKGCH motif is found in SirA^10^. As indicated above, the heme lyase CcmI is essential for maturation of the nitrite reductase, NrfA, of Shewanella^29^. Less is known about the complete maturation of the *Shewanella* sulfite reductase, SirA. SirA is an octaheme c-type cytochrome that is predicted to be the catalytic subunit of the sulfite reductase SirACD, and appears to represent a siroheme-independent class of sulfite reductases^10^.

In this paper we describe the role of CcmI, SirG and SirEFG in the maturation of the sulfite reductase. Contrary to what has been shown for other c-cytochromes of *S. oneidensis*, our results indicated that both the Ccm and Sir systems are essential for proper sulfite reductase maturation. In addition, we identify a novel transcriptional regulator, SirR, required for expression of the sulfite reductase, SirA. It has been shown that sulfite reduction is regulated by the cyclic AMP receptor protein (CRP)^35^, yet it remains unknown if this occurs via direct transcriptional regulation of the sir operon. Here we show that CRP controls expression of SirR, which in turn regulates expression of the sulfite reductase operon.

## Materials and Methods

### Bacterial strains and growth conditions

Bacterial strains and plasmids used in this study are listed in Table 1. Lysogeny Broth (LB) medium was routinely used for aerobic growth of *S. oneidensis* MR-1 and *E. coli* strains. Anaerobic cultures of *S. oneidensis* MR-1 strains were grown in minimal medium (63 mM HEPES pH 7.4, Trace Metals & Minerals) supplemented with 50 mM lactate and 0.02% casamino acids^36^. Electron acceptors were used at 10 mM unless noted otherwise. Growth and reduction of sulfite (10 mM), thiosulfate (5 mM), and tetrathionate (3 mM), was performed anaerobically in a Coy anaerobic chamber using biometer flasks with 10 ml of 40% KOH in the sidearm to trap H_2_S^10^. Chloramphenicol (20 µg/ml), kanamycin (25 µg/ml), and gentamycin (25 µg/ml), were added as appropriate.

**Table 1:** List of strains and plasmids used in this study.

Strain	Description	Source
MR-1	Lake Oneida *S. oneidensis* isolate	Venkateswaran *et al*.^60^
SR1207	MR-1 ∆SO_0479 (*sirA*)	Shirodkar *et al*.^10^
∆*nrfA*	MR-1 ∆*nrfA*	This Work
SR1518	∆*sirA* containing pTC1	This Work
SR1550	MR-1 ∆SO_0265 (*ccmI*)	This Work
SR1576	∆*ccmI* containing pTC2	This Work
SR1566	MR-1 ∆SO_0476 (*sirH*)	This Work
SR1570	∆*sirH* containing pTC3	This Work
SR1542	MR-1 ∆SO_0477-8 (*sirEF*)	This Work
SR1548	∆*sirEF* containing pTC4	This Work
SR1593	MR-1 ∆SO_0482 (*sirG*)	This Work
SR1592	∆*sirG* containing pTC5	This Work
SR1594	∆*ccmI* ∆SO_0482 (*sirG*)	This Work
SR1595	∆*ccmI*∆*sirG* containing pTC2	This Work
SR1596	∆*ccmI*∆*sirG* containing pTC5	This Work
EC100D+	*E. coli* EC100 derivative, pir+	Epicenter Technologies
β2155	pir::RP4, KmR	Dehio *et al*.^38^
pER21	R6K *ori*, Gm^R^, *sacB*, *lacZ α*-fragment	Shirodkar *et al*.^10^
pJBC1	Cloning and sequencing vector, Cm^R^	This Work
pTRG	Bacterial two-hybrid target plasmid	Agilent Technologies
pBT	Bacterial two-hybrid bait plasmid	Agilent Technologies
pTC1	SO_0479_N589C_ in pJBC1	This Work
pTC2	SO_0265 (*ccmI*) in pJBC1	This Work
pTC3	SO_0476 (*sirH*) in pJBC1	This Work
pTC4	SO_0477-8 (*sirEF*) in pJBC1	This Work
pTC5	SO_0482 (*sirG*) in pJBC1	This Work
pBTH1	SO_0479 (*sirA*) in pTRG	This Work
pBTH2	SO_0479 (*sirA*) in pBT	This Work
pBTH3	SO_0482 (*sirG*) in pTRG	This Work
pBTH4	SO_0482 (*sirG*) in pBT	This Work
pBTH5	SO_0265 (*ccmI*) in pTRG	This Work
pBTH6	SO_0265 (*ccmI*) in pBT	This Work

### Generation and complementation of mutants

Chromosomal deletions of *sirH, sirEF, sirG* and *ccmI* were generated using the suicide vector pER21^37^. Approximately 1 kb DNA fragments that flank these genes were amplified by PCR and Phusion polymerase (New England BioLabs). The internal primers were engineered to include restriction enzyme sites and allow ligation of the amplified fragments (see Table 2 for primer sequences). The ligated fragments were cloned into the SmaI digested pER21, and the recombinant plasmids were transferred into *S. oneidensis* MR-1 cells by conjugation from *E. coli* β2155^38^. Mutants were isolated following sucrose selection as described previously^39^ and chromosomal deletions were confirmed by PCR. To complement the mutants, DNA fragments that carry the respective genes were generated by PCR using Phusion polymerase (New England BioLabs). Amplified fragments were cloned into pJBC1^37^ then transferred from *E. coli* β2155^38^ to *S. oneidensis* MR-1 strains by conjugation.

**Table 2:** List of primer used in this study.

Primer	Sequence
Chromosomal Deletions Primers	
265dUF	CTGTTCTGCGATGCTGAC
265dUR	TACGAATTCCATCTACAATAGCGCTAAGTC
265dDF	TACGAATTCCACTTTAGTTTGCATAAAAGCG
265dDR	GTCCAGTTACGGAATGCAC
476dUF	GATTCTTCCCAAGCAATTGC
476dUR	TACGAATTCTCCCGTAAAGCACGGCG
476dDF	TACGAATTCTATTCAAACTGATCGAGTCGC
476dDR	ATCCCGCACCTCAAAACAAC
477dUF	GGCGATAAAGTCAATTTCACG
477dUR	GATCGGATCCGGATTAACCCATGAAATCTTAC
478dDF	GATCGGATCCATTAGTTAACGATTTAC
478dDR	CGTCTTAGAGTGACGTGAACGTC
482dUF	CTGGCGCATGTCAGGAAG
482dUR	GACTGGATCCGAGTGCGATGGTGAACCC
482dDF	GACTGGATCCCGCGATGCCATCAATAACGC
482dDR	GAGCGACTACTGCGACTAACG
**Complementation Primers**	
265cF	TGCGCTAAGCCAAGACTTAG
265dDR	GTCCAGTTACGGAATGCAC
476cF	AAGCATATCACATATGATGAAATCTTACACAGCCAAGC
476cR	CATTTACCGCCGTGCTTTAC
477cF	GTAAGATTTCATGGGTTAATCC
478cR	TCCTAATTTATCATTAGTT
482cF	AAGCATATCACATATGGCATGGCAAGTTTAGGGTTC
482cR	GGCTAGCGACTTGATTAATATC
**Site Directed Mutagenesis Primers**	
479subF	CGGCTTCTGACCACGACGTAACTGAATGCAAAGGTTGTCATAGCCAGTTCCAATC
479subR	GATTGGAACTGGCTATGACAACCTTTGCATTCAGTTACGTCGTGGTCAGAAGCCG
**Bacterial Two-Hybrid Primers**	
TRGCMIEF	GCATCAGAATTCCAACATTTAGGTGCCTTTGAAAATATAGGC
TRGCMIXR	GATCTTACTCGAGTTGTACTTGAGTATCCAGTACTAAGTTTGCGG
BT479BF	CCAGCGGGATCCGCTAAATCGGATGGTAAAGTG
BT479XR	CCGATCTCCTCGAGCATTTTAGCGTTGTAGCCATTACC
TRG482BF	GCGGCCGGATCCGGACGTTATAGCGATTGG
TRG482BR	CTGGCGCTCGAGATATCCACTTTCATTCAATTTTATTTG

### Site directed mutagenesis of SirA

The QuikChange II Site-Directed Mutagenesis Kit from Stratagene (Agilent Technologies, Inc., Santa Clara, CA) was used to generate a mutation in the atypical heme-binding site of SirA. The primers extended 26 bases upstream and downstream of the corresponding N_589_ codon in SirA (see Table 2 for primer sequences). The N_589_ codon, AAC, was changed to TGC resulting in an N_589_C substitution in the translated sequence. The mutagenized fragment was amplified by PCR using PfuUltra HF DNA polymerase and the base substitution was confirmed by DNA sequencing (Eurofins MWG Operon, Huntsville, AL). The mutagenized *sirA* and its native promoter were cloned into the *S. oneidensis* expression plasmid pJBC1^37^. The resulting plasmid was transferred from *E. coli* β2155^38^ into ∆*sirA* by conjugation.

### Protein detection by western blot

Antibodies against SirA peptides (N′- CDGSWGAHGPRYTQKRLD and N′- CHGPQYEKWRRSRHSK) were generated and affinity purified by Biomatik corp. (Cambridge, Ontario). Strains of *S. oneidensis* were incubated anaerobically in basal medium supplemented with 50 mM lactate, 0.02% casamino acids and 10 mM sodium sulfite for 24 hours, unless otherwise indicated. Cells were pelleted by centrifugation and aliquots were used for determination of protein concentrations or resuspended directly in SDS loading buffer and boiled for 10 min^40^. Proteins were separated on 10% sodium dodecyl sulfate-polyacrylamide gels and transferred to polyvinylidene difluoride membrane (Thermo Scientific, Rockford, IL). The membranes were incubated with SirA antibodies and developed using Supersignal West Pico Chemiluminescent substrate (Thermo Scientific, Rockford, IL). Blots were imaged using a FOTO/Analyst Luminary FX Workstation (FOTODYNE Inc., Hartland, WI). Protein concentrations were determined using the Coomassie Plus Protein Assay kit (PIERCE, Rockford, IL).

### Detection of sulfite and nitrite reductase activities in native polyacrylamide gels

*S. oneidensis* wild type and mutant strains were grown anaerobically in basal medium supplemented with 50 mM lactate, 0.02% casamino acids and 10 mM sodium sulfite or 0.5 mM potassium nitrite as electron acceptors. Cell extracts were prepared using B-PER lysis reagent (PIERCE, Rockford, IL). Protein concentrations were determined using the Coomassie Plus Protein Assay kit and 50 μg total protein was separated on 10% native polyacrylamide gels. Enzyme activities were assayed essentially as previously described^41^. Gels were transferred to a Coy anaerobic chamber (Coy Laboratory Products, Grass Lake, MI) and stained for 20 min with reduced methyl viologen [38.9 mM methyl viologen dichloride and 57.4 mM sodium hydrosulfite in 10 mM Tris pH 7]. 10 mM of Na_2_SO_3_ or KNO_3_ was added and the gels were incubated further until bands of clearing, indicating reduction of the electron acceptors, were observed. Gels were imaged using a Kodak DC290 digital imaging system (Eastman Kodak, Rochester, NY).

### Sulfite reduction assays

Reduction of sulfite was performed anaerobically in a Coy anaerobic chamber using biometer flasks as described previously^10^. The flasks contained 100 ml of deoxygenated minimal medium supplemented with 50 mM lactate, 0.02% casamino acids and 10 mM sodium sulfite as the sole electron acceptor. Chloramphenicol (20 μg/ml) was added as appropriate. The side arm of the flasks contained 10 ml of 40% KOH to trap H_2_S^42^. Overnight cultures of *S. oneidensis* strains grown in LB were used as inocula. Cultures were sampled to measure H_2_S and SO_3_^−2^ every 12 to 24 hrs for up to 120 hrs. H_2_S was measured using the mixed diamine assay^43^ with modification. Briefly, 0.5 ml of the KOH trap was transferred to 25 ml of dH_2_0 and 1 ml of mixed diamine reagent (20 g *N, N-*dimethyl-*p-*phenylenediamine sulfate, 30 g ferric chloride (FeCl_3_·6H_2_0) in 500 ml of 50% hydrochloric acid) was added. The color was allowed to develop for 20 minutes and the absorbance was measured at 670 nm. Hydrogen sulfide concentrations were determined using sodium sulfide as a standard. Sulfite concentrations were determined using the fuchsin assay as described previously^44^ using sodium sulfite as the standard. Briefly, 100 μl of 1.18 mM fuchsin dye (dissolved in 2.25 N H_2_SO_4_) was added to 20 μl of sample mixed with 870 μl of water. Following 10 min incubation at room temperature, 10 µl of formalin was added, and the absorbance was measured at 570 nm.

### Nitrite reduction assay

Reduction of nitrite was performed anaerobically in a Coy anaerobic chamber. Serum vials containing deoxygenated basal medium supplemented with 50 mM lactate, 0.02% casamino acids, 0.5 mM potassium nitrite, were inoculated with *S. oneidensis* cultures grown overnight in LB. Cultures were sampled every hour and nitrite concentrations were measured using N-(1-naphthyl)ethylenediamine dihydrochloride and sulfanilic acid. The mixture was incubated for 10 min, and the absorbance was measured at 540 nm.

### β-galactosidase assays

The DNA fragments upstream of *sirA* and *sirR* were amplified by PCR and Phusion polymerase (New England Biolabs), digested with HindIII and BamHI, then cloned into pMC10^45^. Following transformation of *E. coli* β2155, the resulting plasmids were transferred into *S. oneidensis* wild type and mutant strains by conjugation. β galactosidase activity was determined using ONPG (9 mg of ONPG in 10 ml of 1 mM MgCl_2_ and 0.1 mM β-mercaptoethanol) as described previously^46^.

### Quantitative reverse-transcription PCR

Wild-type or mutant cells were grown overnight under aerobic conditions in triplicate. Cultures were pelleted, and RNA was isolated using the TRIzol Plus RNA Purification System (ThermoFisher) according to manufacture instructions. Chromosomal DNA was removed by treatment with DNaseI, cleaned up and total RNA quantified on a Qubit 4 (ThermoFisher). cDNA was generated with the High-Capacity RNA-to-cDNA kit and Realtime-PCR was carried out on a CFX96 (BioRad) with the SsoAdvanced Universal SYBR Green Supermix (Biorad). Gene specific primers were synthesized by IDT Technologies, and primer specificity was confirmed by melt curve analysis followed by agarose gel electrophoresis, no template and no reverse transcriptase were performed as appropriate. Relative expression was determined by the delta-delta-Ct method from three biological replicates assayed in technical triplicates.

### Bacterial two-hybrid

Genes SO_479, SO_482 and SO_265 were PCR amplified from *S. oneidensis* MR-1 chromosomal DNA with Phusion DNA polymerase (New England BioLabs, Ipswich, MA) and purified with the IBI Gel/PCR DNA fragment extraction kit (IBI Scientific, Dubuque, IA) (see Table 2 for primer sequences). The SO_479 (*sirA*) DNA fragment was digested with BamHI and XhoI and ligated into pTRG. Similarily, SO_482 (*sirG*) and SO_265 (*ccmI*) DNA fragments were digested with EcoRI and XhoI and then ligated pBT. The resultant plasmids were transformed into *E. coli* XL1-Blue MRF´ Kan and screened on LB-chloramphenicol (for pBT transformants) or LB-Tetracycline (for pTRG transformants). Colonies that contained the appropriate vectors were confirmed by PCR. The vector pairs were then co-transformed into the BacterioMatch II two-hybrid reporter strain. Transformed cells were plated on M9 non-selective and selective media that contained 3-amino-1,2,4-triazole (3AT) supplemented with chloramphenicol and tetracycline. Additional vectors and reporter strains were also generated with the fragments switched (i.e. *sirA* in pBT and 482 and *ccmI* in pTRG). To confirm interactions between the proteins, reporter strains were grown overnight in M9 + His broth and serially diluted (up to 10-7). 10 µl were spotted on M9, M9 + 3AT and M9 + 3AT agar supplemented with 12.5 µg streptomycin per mL and allowed to grow at 37 °C for up to 48hrs.

## Results

### CRP regulates sulfite reduction via the transcriptional regulator SirR

**Figure 1:**
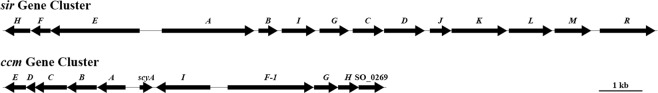
Arrangement of the *sir* and *ccm* gene clusters on the *S. oneidensis* chromosome. Letters correspond to the *sir* or *ccm* gene names unless indicated otherwise.

**Figure 2:**
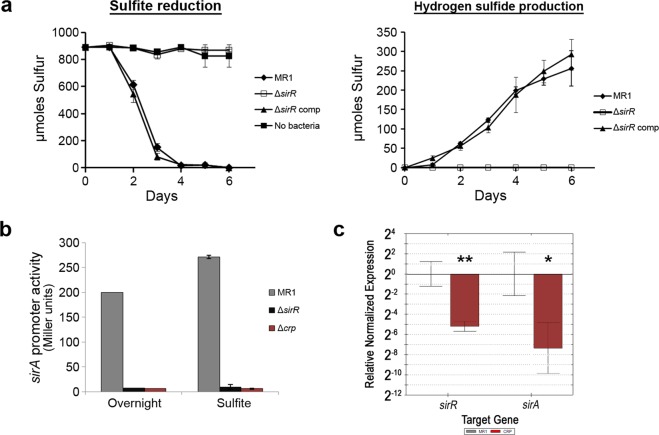
SirR regulates sulfite reduction via regulation of *sirA* expression. (**a**) Sulfite reduction is indicated by loss of sulfite (SO_3_) and accumulation of hydrogen sulfide (H_2_S). Loss of *sirR* completely abolished sulfite reduction. Complementation of *sirR* restored sulfite reduction to wild type levels. (**b**) Activity of the *sirA* promoter was measured by *lacZ*-promoter fusions. Beta-galactosidase activity was measured after overnight aerobic growth or anerobic growth with sulfite as the sole electron acceptor. Both *sirR* and *crp* were required for expression of the sulfite reductase operon, as indicated by loss of *lacZ* expression in the Δ*sirR* and Δ*crp* mutants. **(c**) Expression of *sirR* and *sirA* in the wild-type MR-1 and Δ*crp* mutant. CRP was required for wild-type level expression of *sirR* and *sirA*, which indicated that CRP was required for optimal expression of *sirR* as well as *sirA*. *p < 0.05, **p < 0.005, ANOVA.

The genes involved in anaerobic sulfite reduction in *S. oneidensis* MR-1, *sirAGCD*, are located within a cluster of genes designated the *sir* locus (Fig. 1). SO_0490, which we designated *sirR*, lies just downstream of the *sir* locus and encodes a protein that belongs to the ToxR family of transcriptional activators. To determine if SirR regulates genes required for anaerobic growth, a *sirR* (∆*sirR*) mutant was generated and tested for anerobic respiration with diverse electron acceptors utilized by the wild type *S. oneidensis* MR-1. The ∆*sirR* mutant was deficient in sulfite reduction but grew with all other electron acceptors tested. Complementation of the ∆*sirR* mutant restored sulfite reduction and H_2_S production to wild type levels (Figs. 2a and S1). To demonstrate that SirR regulates the expression of the *sirAIGCDJKLM* operon, a *sirA* promoter-*lacZ* fusion was generated. Expression of the *sirA* operon was assessed, by way of promoter activity, in the MR-1 parent strain, the ∆*sirR* mutant, and as a negative control, the ∆*crp* mutant. Expression of the *sirA* operon was eliminated in both the ∆*sirR* and ∆*crp* mutants (Fig. 2b). These results indicated that both SirR and CRP were required for sulfite reduction, yet it was unknown whether these proteins regulated *sirA* expression independently or as part of a regulatory cascade.

CRP is known to regulate the expression of many genes involved in anaerobic respiration, and sequences that match the *E. coli* CRP-binding site (TGTGA------TCACA) were identified upstream of *sirR* (TGAGT------TCACA). To determine if CRP regulates *sirR* expression, we assessed relative transcripts of *sirR* and *sirA* in the MR-1 wild-type and Δ*crp* mutant. The transcript level of *sirR* was significantly reduced in the ∆*crp* mutant compared to the wild type (p = 0.002), and subsequently sirA transcript was further reduced in the Δ*crp* mutant (p = 0.018) (Fig. 2c). These results indicated that CRP positively regulates the expression of *sirR*, which correlates with previously published transcriptomic data that indicated reduced expression of *sirR* in the ∆*crp* mutant^35^. These results further suggest that CRP likely regulates sulfite reduction via regulation of the sulfite reductase regulator SirR.

### The atypical heme binding motif of SirA is required for sulfite reduction

**Figure 3:**
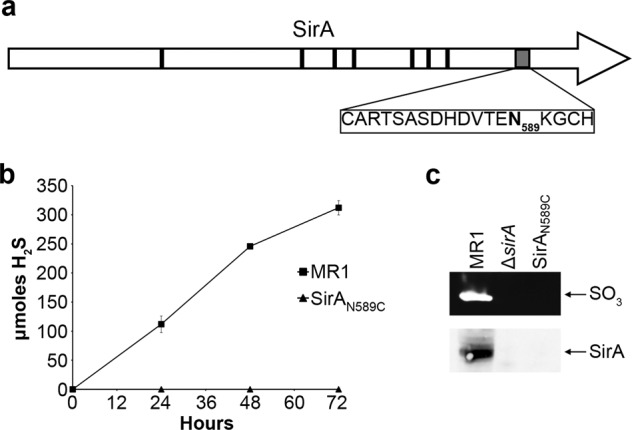
The atypical heme binding site of SirA is required for maturation and stability. (**a**) Locations of typical and atypical heme binding sites of SirA. Black boxes indicate typical CXXCH binding sites, the gray box indicates the atypical CX_12_NKGCH binding site. Asparagine 589 (bolded) was changed to a cystine to restore the atypical binding site to the typical CXXCH motif. (**b**) *Shewanella* that expressed the wildtype or mutated SirA were grown anaerobically on sulfite. The SirA_N589C_ mutant was unable to reduce sulfite, as indicated by lack of hydrogen sulfide production. (**c**) Whole cell extracts from the wildtype, Δ*sirA* and SirA_N589C_ strains were tested for sulfite reductase activity and the SirA protein. Neither Δ*sirA* nor SirA_N589C_ cell extracts had an active sulfite reductase or a band that reacted with antibodies directed against SirA. Full-length blots/gels are presented in Supplementary Fig. S3.

The *S. oneidensis* sulfite reductase SirA belongs to the MccA family of *c*-type cytochromes and in addition to typical CXXCH heme binding sites, contains a CX_12_NKGCH motif predicted to bind heme^47^ (Fig. 3a). A CX_12_AKGCH motif was found to be essential for the activity of the *W. succinogenes* sulfite reductase^22^. We hypothesized that this atypical heme-binding motif is essential for the *S. oneidensis* SirA sulfite reductase activity as well. To test this, we generated a SirA mutant, SirA_N589C_, where the asparagine in the CX_12_NKGCH motif was replaced with a cysteine to generate a typical heme-binding site (CX_12_CKGCH). The ability of SirA_N589C_ to restore sulfite reductase activity to the ∆*sirA* mutant was tested by measuring H_2_S production with sulfite as the sole electron acceptor. Our results indicated that SirA_N589C_ was completely deficient in sulfite reduction (Fig. 3b). Furthermore, we did not detect sulfite reductase activity by SirA_N589C_ in native polyacrylamide gels when methyl viologen was used as the electron donor. This deficiency appears to be due to loss of protein as evidenced by the lack of a band that reacted with peptide antibodies generated against SirA (Fig. 3c). These results support the prediction that the atypical CX_12_NKGCH site is essential for SirA activity, as was observed in its *W. succinogenes* counterpart.

### The Ccm and Sir systems are required for proper maturation of the sulfite reductase SirA

**Figure 4:**
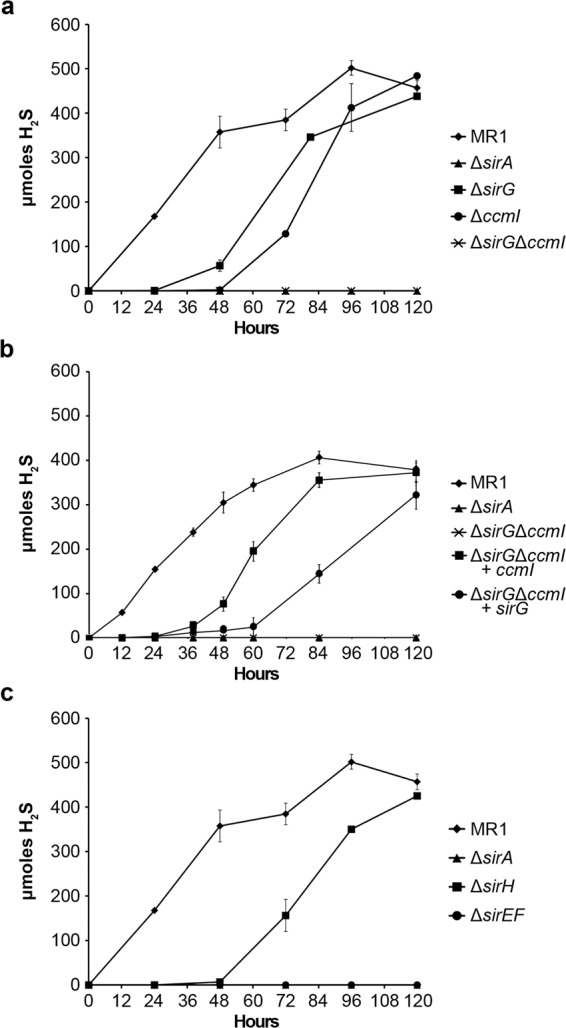
Cytochrome maturation and heme lyase components are required for optimal sulfite reduction. Wildtype and mutant *S. oneidensis* were grown anaerobically with sulfite as the sole electron acceptor. Sulfite reduction was indicated by the production of hydrogen sulfide (H_2_S). (**a**) Loss of either heme chaperone, SirG or CcmI, resulted in a lag in sulfite reduction, likely due to a reduction in functionally active reductase. Sulfite activity was abolished when both *sirG* and *ccmI* were deleted. (**b**) Sulfite reductase activity of the Δ*sirG*Δ*ccmI* double mutant was partially restored, similar to that of the respected single mutant, when complemented with either *sirG* or *ccmI*. (**c**) Loss of the heme lyase components SirH or SirEF resulted in decreased sulfite reduction. The Δ*sirH* mutant exhibited a 48-hour lag in sulfite reduction compared to wildtype, whereas the Δ*sirEF* mutant lacked any sulfite reduction. The Δ*sirA* mutant served as a negative control for sulfite reduction in all assays.

Based on studies of heme maturation systems in other bacteria, such as *E. coli* and *W. succinogenes*^22,30,31,33,47,48^, we hypothesized that heme ligation to the atypical CX_12_NKGCH motif of SirA would require a specific and dedicated heme lyase complex. To identify the proteins involved in maturation of SirA, we first tested the role of CcmI and SirG in sulfite reduction. Previous studies that investigated the role of CcmI in maturation of the nitrite reductase NrfA, indicated that loss of CcmI may result in a partial, but incomplete, defect in SirA enzyme activity^29^. Accordingly, we found that mutants that lack either SirG or CcmI were able to reduce sulfite, although a lag phase of 24 hr (SirG) and 48 hr (CcmI), with respect to wildtype, was observed before H_2_S production was detected (Fig. 4a). However, a mutant that lacked both SirG and CcmI was completely deficient in sulfite reduction, which suggested that SirG and CcmI may be able to partially compensate for loss of one another (Fig. 4a). Introduction of *sirG* into the ∆*sirG*∆*ccmI* mutant partially restored sulfite reduction, with reduction rates similar to that of the single ∆*ccmI* mutant. Similarly, introduction of *ccmI* into the ∆*sirG*∆*ccmI* mutant resulted in sulfite reduction rates similar to that of the *∆sirG* mutant (Fig. 4a,b). These results indicated that, contrary to nitrite reduction where CcmI was essential, neither CcmI nor SirG were essential for sulfite reduction. Both proteins appear to have a partially redundant function, and complete loss of sulfite reduction was observed only when both proteins were absent.

To confirm that both SirG and CcmI directly interact with the apo-SirA during maturation, bacterial two-hybrid analysis was performed. Constructs were generated to assess the interaction between SirA and SirG, and between SirA and CcmI. The bacterial two-hybrid assay indicated that both SirG and CcmI interact with SirA independently of one another and in the absence of other components of the *S. oneidensis* Ccm and Sir maturation systems (Supplementary Fig. S2). Complimentary assays were performed in which SirA served as both the bait (pBT) or the target (pTRG) protein. The results obtained were the same regardless of the orientation of the two-hybrid assay, and further supported the observation that both SirG and CcmI are involved in direct maturation of the SirA sulfite reductase.

Located within the *sir* gene cluster, are *sirEFH*. SirEF are similar to the NrfEF cytochrome *c* synthetase and thiol-oxidoreductase that are required for heme ligation to the CXXCK site in NrfA (31). Interestingly, previous work found that SirE is not required for nitrite reduction in *S. oneidensis*^29^. The third gene in the *sirEFH* operon, *sirH*, encodes a protein similar to thioredoxin-like proteins of the TlpA- DsbE- ResA family^49^. It is predicted to be a periplasmic protein with 63% similarity to the protein encoded by SO_0269, which lies downstream of *ccmH* (Fig. 1). SirH also shares similarity with *P. aeruginosa* thioredoxins (NP_251167.1 and NP_249644.1) with 54% and 49% similarity, respectively. To determine if SirEFH were required for maturation of a functional sulfite reductase, a ∆*sirEF* double mutant and a ∆*sirH* single mutant were generated and tested for the ability to reduce sulfite. Deletion of *sirEF* resulted in complete loss of sulfite reduction (Fig. 4c). Complementation of the ∆*sirEF* mutant restored sulfite reduction to wild type levels. In contrast, a mutant that lacked *sirH*, which appears to form an operon with *sirEF*, was able to reduce sulfite at a rate similar to wild type after a lag period of 48 hr (Fig. 4c). This delay in reduction may be due to inefficient maturation of the sulfite reductase, suggesting that SirH is important, but is not essential, for maturation of SirA.

### Sulfite reductase activity and protein stability

**Figure 5:**
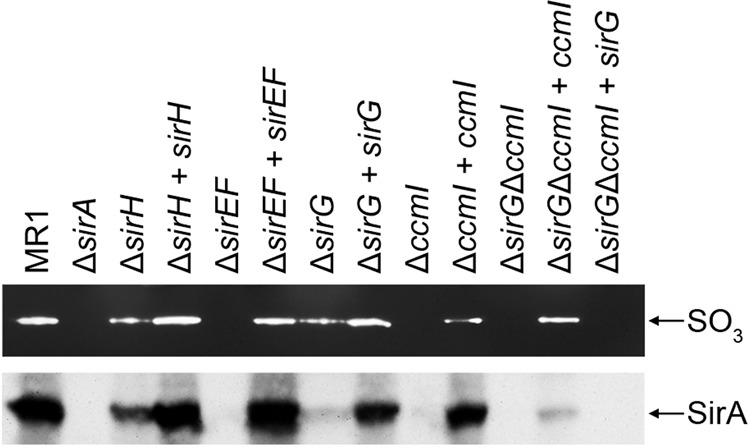
Sulfite reductase activity and protein levels of wild-type, mutant, and complemented mutant cell extracts. Upper panel. Sulfite reductase activity was indicated by bands of clearing. No activity was observed in extracts from Δ*sirA*, Δ*sirEF*, Δ*ccmI* or Δ*ccmI*Δ*sirG*. Reduced activity was observed in cell extracts from Δ*sirH* and Δ*sirG*. Complementation restored reductase activity to wildtype levels, except with Δ*ccmI*, which partially restored activity. Lower panel. Western blot analysis of the cell extracts was performed using antibodies against SirA. Reactive bands that corresponded to SirA are indicated. Reduced SirA was detected in extracts from Δ*sirH* and Δ*sirG* compared to wildtype and was absent in extracts from Δ*sirA*, Δ*sirEF*, Δ*ccmI* and Δ*ccmI*Δ*sirG*. Complementation restored SirA levels similar to that of the wildtype or corresponding single mutants, in the case of the Δ*sirG*Δ*ccmI* mutant. Full-length blots/gels are presented in Supplementary Fig. S5.

To further elucidate the role of the heme lyase proteins on maturation of the terminal sulfite reductase, sulfite reductase activity was measured in wildtype and mutant strains. *S. oneidensis* strains were grown for 24 hr with SO_3_^−2^ and then tested for sulfite reductase activity on native polyacrylamide gels using methyl viologen as the sole electron donor. Reductase activity was indicated as a band of clearing in the gel. As expected, ∆*sirA* was completely deficient in sulfite reductase activity (Fig. 5). Cell extracts from the ∆*sirH* and ∆*sirG* mutants exhibited decreased sulfite reductase activity, which was restored to wild type levels by complementation of the mutation. Cell extracts from the ∆*sirEF* mutant completely lacked sulfite reductase activity (Fig. 5), which further supported the hypothesis that SirEF are essential for maturation of the sulfite reductase. Sulfite reductase activity was barely detectable in *∆*sirG and completely absent in ∆*ccmI* grown for 24 hr, further substantiating the sulfite reduction results presented in Fig. 4 and indicating that both SirG and CcmI have a role in sulfite reducatase activity at this time point. Cell extracts of the ∆*ccmI*∆*sirG* double mutant completely lacked sulfite reductase activity (Fig. 5). Moreover, introduction of *ccm*I, but not *sirG*, into the ∆*ccmI*∆*sirG* double mutant restored sulfite reductase activity (Fig. 5). These results support our findings that CcmI has a more prominent role in sulfite reductase maturation than SirG, although neither CcmI or SirG can fully support wild type levels of SirA maturation. Together these observations indicated that sulfite reduction by intact cells is proportional to sulfite reductase activity of cell extracts, and further suggested that the observed sulfite reduction deficiency in the mutants was not due to loss of other *c*-type cytochromes that are part of the electron transport chain that leads to sulfite reduction.

The observed decrease or absence of sulfite reductase activity in cell extracts of the *ccmI* and *sirEF*, *sirG*, and *sirH* mutants may be due to decreased catalytic activity or reduced protein levels. To distinguish between these two possibilities, we assessed SirA levels in cell extracts from the wild type strain and mutants using peptide antibodies generated against SirA. A reactive band of 72 kDa that corresponded to the predicted size of SirA was detected in the wild type and to a lesser extent in the ∆*sirH* and *∆sirG* cell extracts (Fig. 5). This band was absent in cell extracts from the ∆*sirEF* and ∆*ccmI* mutants and was restored by complementation. Similar to the results obtained for enzyme activity, cell extracts from the ∆*sirG*∆*ccmI* double mutant lacked SirA, presumably as a result of protein degradation. Cell extracts of the ∆*sirG*∆*ccmI* mutant complemented with *ccmI*, had a faint band that reacted with SirA antibodies; however, the band was absent in cell extracts when the double mutant was complemented with only *sirG* (Fig. 5). These results indicated that the sulfite reduction deficiency observed in the cytochrome *c* maturation mutants was due to decreased protein levels, most likely due to instability and degradation of the unmatured apoprotein.

### The Sir system is exclusive to the maturation of the SirA reductase

**Figure 6:**
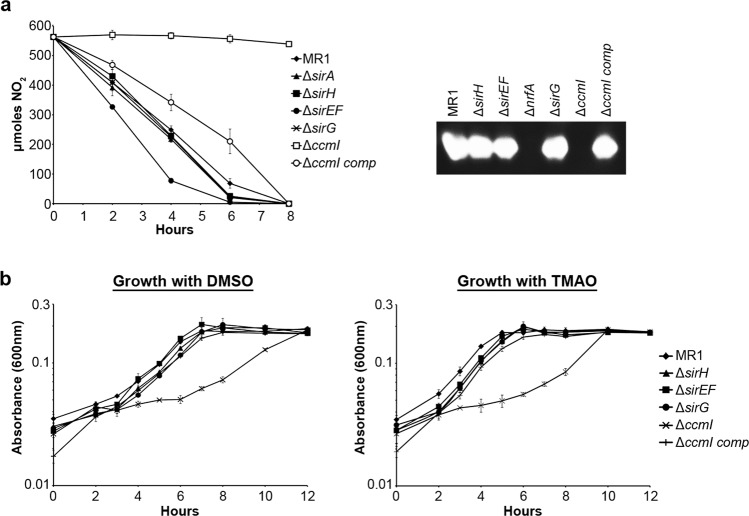
The *sir* cytochrome maturation genes are not involved in maturation of the NrfA nitrite reductase. (**a**) Nitrite reduction was not significantly decreased in any of the *sir* mutants, however the Δ*sirEF* mutants reduced nitrite faster than the wildtype. As anticipated, Δ*ccmI* was unable to reduce nitrite and reduction was restored by complementation. Enzymatic activity of the nitrate reductase was not affected in any of the *sir* mutants. There was no nitrite reductase activity in the cell lysates of Δ*nrfA* or Δ*ccmI*. (**b**) Δ*sirH*, Δ*sirEF* and Δ*sirG* were all able to grow on DMSO or TMAO similar to wildtype. The Δ*ccmI* mutant exhibited a lag in growth with either DMSO or TMAO, which was restored by complementation. Full-length gel is presented in Supplementary Fig. S4.

Previous reports indicated that the SirEFH complex is not required for maturation of c-cytochromes that possess only typical CXXCH heme-binding sites^28^ and that SirE is not required for nitrite reductase activity^29^. However, it remained unclear what role, if any, SirG plays in the maturation of any of these other c-cytochrome reductases. As both CcmI and SirG were involved in maturation of the sulfite reductase, which possesses both typical and atypical heme-binding sites, we first determined if SirG was involved in maturation of the nitrite reductase, NrfA, which also possesses an atypical, albeit unique, heme-binding site. Nitrite reduction assays and direct measurement of enzymatic activity suggest that SirG is not required for maturation of NrfA and that SirG is unable to compensate for loss of CcmI, with respect to NrfA maturation (Fig. 6a). We also assessed the role of SirG in maturation of two c-cytochromes that only have typical CXXCH sites, the DMSO and TMAO reductases. Growth with DMSO or TMAO as the sole electron acceptor was unaffected by loss of any of the *sir* genes. As expected, loss of CcmI resulted in decreased growth with either DMSO or TMAO. Interestingly, following a long lag phase, compared to wild type, the Δ*ccmI* mutant was able to grow to wild type levels with both DMSO or TMAO as the sole electron acceptor (Fig. 6b). These results confirmed that SirEFGH are required for optimal maturation of the sulfite reductase, but do not have an appreciable role in the maturation of other c-type cytochromes.

## Discussion

Maturation of *c*-type cytochromes requires specialized systems for the covalent attachment of heme *b* to CXXCH motifs of apocytochromes. In bacteria, System I (Ccm) or System II (Ccs) is required for holocytochrome *c* maturation and involves an apocytochrome *c* chaperone and a cytochrome *c* synthetase. In bacteria such as *Rhodobacter capsulatus* and *Pseudomonas aeruginosa*, CcmI, CcmH, and CcmF fulfill these roles, while in *Escherichia coli* CcmH appears to be a fusion protein that carries out the functions of both CcmI and CcmH^23,25,50,51^. For *c*-type cytochromes with atypical heme-binding motifs, such as CXXCK found in the nitrite reductase of *E. coli* and other bacteria, specific heme lyases are required for heme attachment to these non-conventional sites. The genome of the metal reducer *S. oneidensis* MR-1 encodes 42 *c*-type cytochromes, many of which serve as terminal reductases during anaerobic respiration and all contain the typical CXXCH motif^16,17^. The System I proteins CcmABCDEFGH were found to be essential for maturation of all *c*-type cytochromes in *S. oneidensis*^28,29^, but none of these proteins were predicted to act as the apocytochrome *c* chaperone component of the heme lyase. Recently, CcmI_*So*_ (SO_0265) was identified as a cytochrome *c* maturation protein required for maturation of the nitrite reductase NrfA^28,29^ and that we predicted functions as an apocytochrome *c* chaperone for maturation of the sulfite reductase SirA. In contrast to an *R. capsulatus ccmI* null mutant that is completely deficient in mature *c*-type cytochromes^52^, an *S. oneidensis ccmI* mutant was able to grow anaerobically similar to the wild type with some electron acceptors, but failed to grow with others^28,29^.

*S. oneidensis* MR-1 possess two c-cytochromes, NrfA and SirA, that in addition to typical CXXCH heme-binding motifs, contain atypical heme-binding sites. The *S. oneidensis* nitrite reductase, NrfA, is a pentaheme *c*-type cytochrome with 4 CXXCH and one CXXCK heme-binding sites, and is similar to the NrfA proteins of *E. coli* and *W. succinogenes*^30,53–55^. Maturation of NrfA in *W. succinogenes* and *E. coli* requires the dedicated heme lyases NrfI and NrfEFG, respectively^30,32,48,56^. Unlike these bacteria, *S. oneidensis* does not appear to have a dedicated heme lyase system for the maturation of its NrfA protein. Although CcmI is essential for the maturation of NrfA, it also participates in the maturation of other *c*-type cytochromes. Furthermore, CcmF is required for maturation of NrfA, yet loss of SirEF has no effect on nitrite reduction. The lack of a dedicated cytochrome *c* synthetase system for maturation of the nitrite reductase in *S. oneidensis*, suggests that CcmFHI may form the heme lyase complex responsible for heme attachment to the CXXCK site. If this is the case, then it further suggests that the same heme synthetase CcmF of *S. oneidensis* is able to ligate heme to apocytochromes *c* with both CXXCH and CXXCK heme-binding motifs.

The *S. oneidensis* sulfite reductase subunit, SirA, is an octaheme *c*-type cytochrome with 7 CXXCH motifs and an atypical CX_12_NKGCH site^10^. Similar atypical sequences have also been identified in the sulfite reductase of *Wolinella succinogenes* and in the MccA family of *c*-type cytochromes^22,47^. In *S. oneidensis*, this site appears to be important for the activity and stability of SirA, and may be the catalytic site of the enzyme. Substitution of the asparagine residue in CX_12_NKGCH with a cysteine, to mimic the typical CXXCH heme-binding motif, completely abolished sulfite reductase activity. Furthermore, we did not detect SirA_N589C_ in Western blots suggesting that the mutation led to instability and degradation of the protein. These findings are similar to previous reports where amino acid substitutions in the heme-binding site or deletion of *ccm* genes lead to instability of the apocytochromes^30,31,57,58^ and ineffective maturation of the NrfA reductase leads to rapid degradation^29^.

To date, maturation of the MccA family of *c* type cytochromes has been studied only in *W. succinogenes*. In this bacterium, a specific heme lyase system is responsible for heme attachment to the CX_15_CH motif^22,33^. Our results described above show that in *S. oneidensis*, *ccmI*_*So*_ is involved in the maturation of the sulfite reductase SirA, which contains an atypical CX_12_NGKCH site. A *ccmI*_*So*_ null mutant produced mature sulfite reductase more slowly than the wild type as indicated by reduction and enzyme assays. We consistently observed a lag phase of 48 hr in sulfite reduction by ∆*ccmI*_*So*_, which suggested that unlike its essential role in the maturation of the nitrite reductase NrfA, CcmI was involved in, but not required for, maturation of the sulfite reductase SirA. ∆*ccmI*_*So*_ was also deficient in DMSO and TMAO respiration, as has been shown previously^28^, but was able to grow to wild type levels after an extended lag phase. The ∆*ccmI*_*So*_ phenotypes were surprising for several reasons. First, all *c*-type cytochromes involved in TMAO, DMSO, and fumarate reduction contain only typical CXXCH heme-binding motifs and a role for CcmI in the maturation of these proteins is unexpected. Second, the non-conventional heme-binding sites in the nitrite and sulfite reductases (CXXCK and CX_12_NGKCH respectively) are different, yet CcmI appears to be involved in the maturation of both proteins, but is only essential for the maturation of NrfA^29^.

In *S. oneidensis*, we have identified SirEFG as a heme lyase that appears to be specific for the maturation of SirA. SirEFG are similar to the *E. coli* proteins NrfEFG that constitute the heme lyase required for maturation of NrfA^30,53^. SirEF, which are similar to cytochrome *c* synthetases (CcmF) and thiol oxidoreductases (CcmH)^59^, appear to be essential for sulfite reductase activity and stability. Our results suggest that SirA requires a dedicated cytochrome *c* synthetase for its maturation, but unlike other *c*-type cytochromes studied to date, it appears to require two apocytochrome *c* chaperones for this process. Single mutants that lack either SirG or CcmI were able to express an active SirA enzyme, albeit at lower levels than the wild type, whereas the double mutant ∆*ccmI*∆*sirG* mutant completely lacked sulfite reductase activity and the SirA protein. Based on these results, we propose that SirEFG along with CcmI constitute a 4-subunit heme-lyase complex that functions in maturation of SirA.

Our results described above, in combination with previously published findings, indicate an unusual cytochrome *c* maturation system in *S. oneidensis*. This bacterium has two predicted heme synthetases, CcmF and SirE and two apocytochromes *c* chaperones, CcmI and SirG. We predict that multiple combinations of these proteins in *S. oneidensis* can form heme lyase complexes depending on growth conditions. For example, CcmI may interact with CcmF and CcmH when nitrite and DMSO are used as electron acceptors. CcmI may also interact with SirEFG when cells are grown with sulfite. Unlike other bacteria studied to date, *S. oneidensis* does not appear to have a dedicated and specific heme lyase complex for each of the heme-binding motifs found in its *c*-type cytochromes. These findings add another twist to the already complex mechanism of cytochrome *c* maturation in bacteria.

## Supplementary information

Supplementary Information.

## Data Availability

No datasets were generated or analysed during the current study.
