# Supplementary material for: Regulation and Maturation of the Shewanella oneidensis Sulfite Reductase SirA
Source: Sci Rep. 2020 Jan 22;10:953. doi: 10.1038/s41598-020-57587-6 (PMC6976685; doi:10.1038/s41598-020-57587-6)
Supplement: Supplementary file 1 — Supplementary Information. [file 41598_2020_57587_MOESM1_ESM.pdf]

Regulation and Maturation of the *Shewanella oneidensis* Sulfite Reductase SirA

Kenneth L. Brockman[#*], Sheetal Shirodkar[§], Trevor J. Croft[‡], Rini Banerjee, Daad A. Saffarini

Department of Biological Sciences, University of Wisconsin – Milwaukee, Wisconsin 53211

[#] Present Address: Medical College of Wisconsin
Department of Microbiology & Immunology

[§] Present Address: Amity University Uttar Pradesh
Amity Institute of Biotechnology

[‡] Present address: University of California Davis
Department of Microbiology and Molecular Genetics

*Corresponding author

Medical College of Wisconsin

Department of Microbiology & Immunology

8701 Watertown Plank Road

Milwaukee, WI 53226

email: kbrockman@mcw.edu

Reduction of sulfur containing compounds

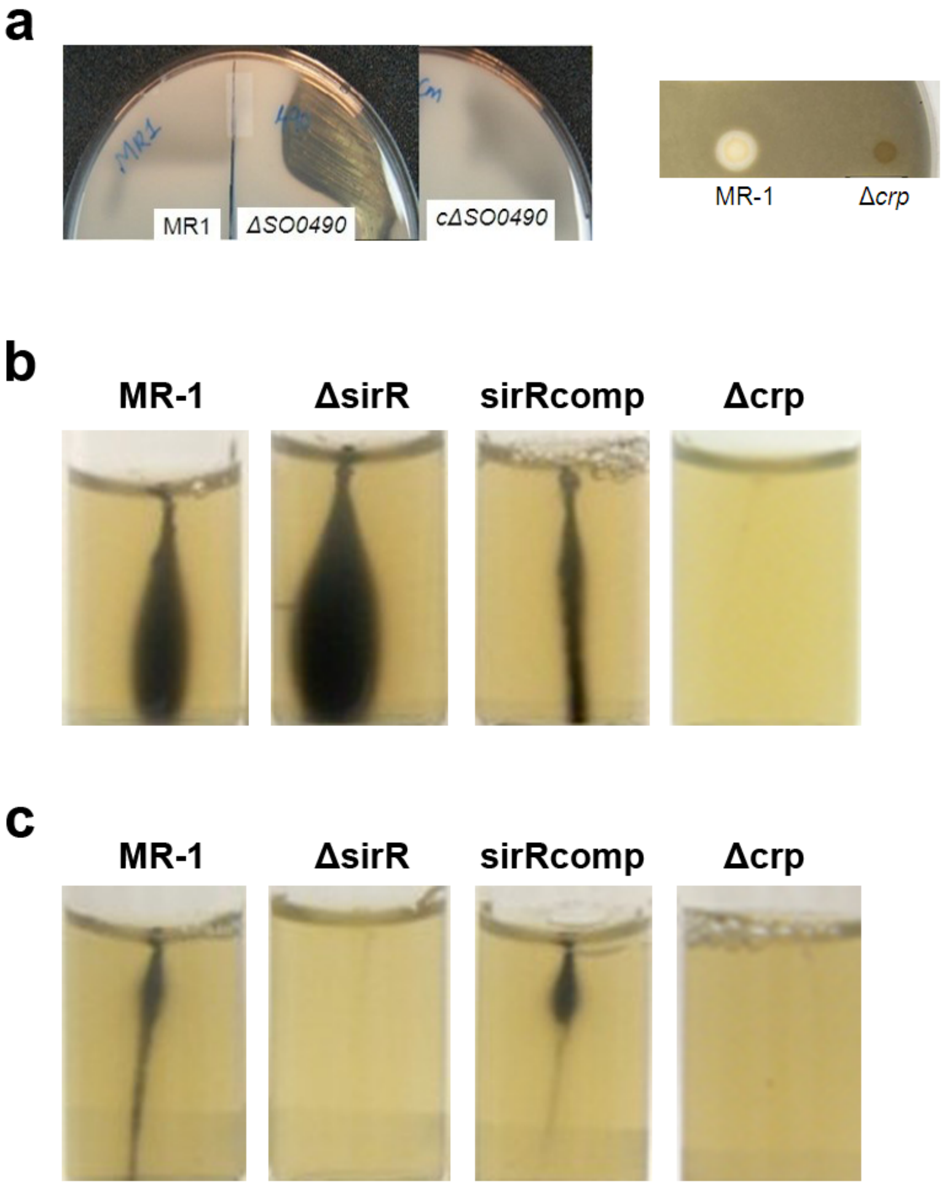

Supplementary Figure S1. Sulfur, thiosulfate and sulfite reduction by MR-1, Δ*sirR*, complemented Δ*sirR* and Δ*crp*. A. Sulfur reduction by S.oneidensis strains. Zones of clearance indicate reduction of sulfur. SirR appears to be a negative regulator of sulfur reduction as seen by the enhanced clearing zone. Complementation of the Δ*sirR* mutant resulted in restoration of wild-type phenotype. B. Thiosulfate (S_2O_3) reduction is indicated by the formation of FeS (black precipitate). The Δ*sirR* mutant was able to reduce S_2O_3 faster than the wild type and complementation restored reduction similar to wild type. C. Sulfite (SO_3) reduction was indicated by the formation of FeS (black precipitate). The Δ*sirR* mutant was able to reduce SO_3 faster than the wild type and the complemented strain restored wild type levels of reduction. The Δ*crp* mutant served as a negative control for all electron acceptors tested.

Bacterial two-hybrid

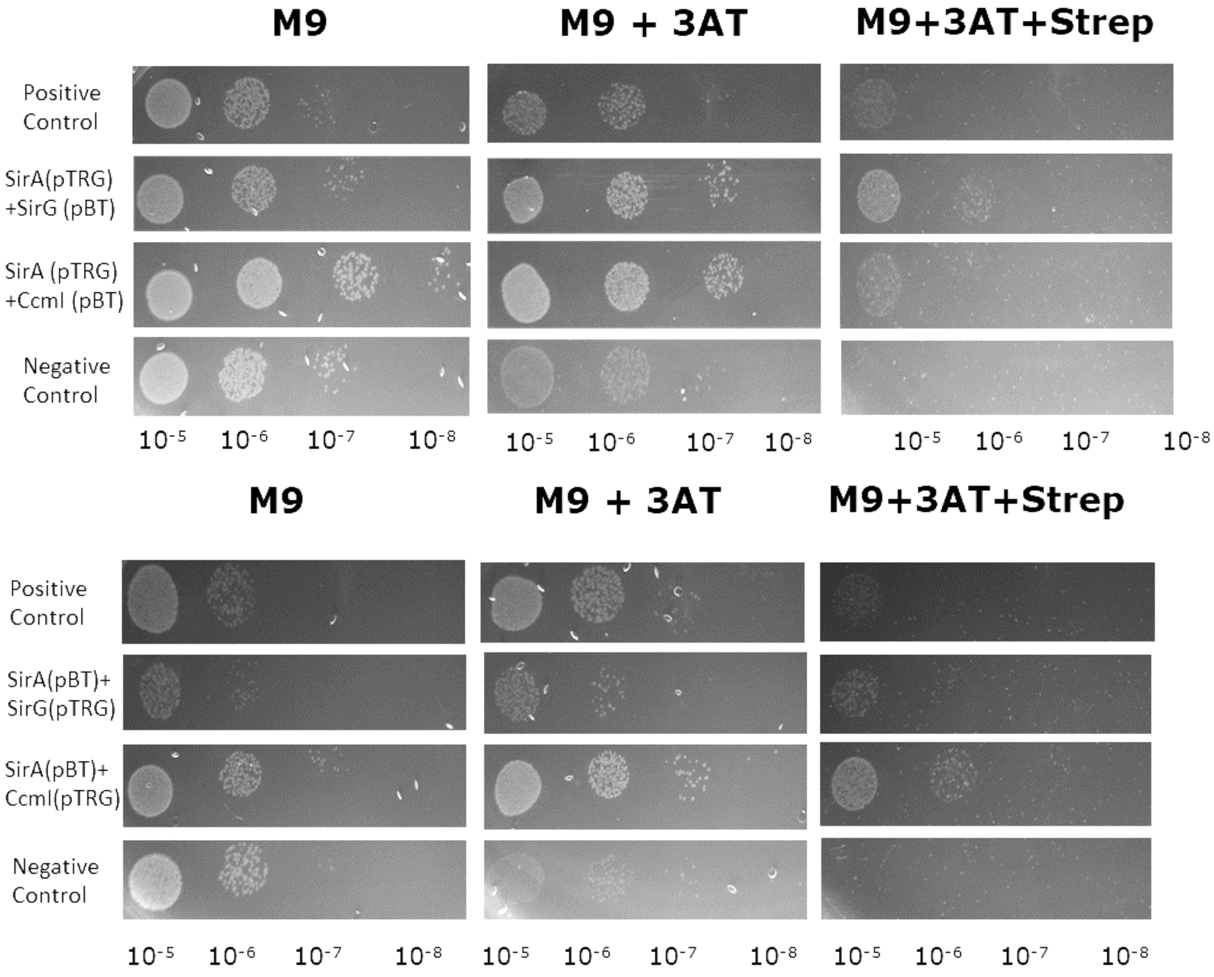

Supplementary Figure S2. The SirG and CcmI heme lyases interact with SirA. A bacterial two-hybrid assay was performed with the BacteriMatch II Two-Hybrid System Kit, according to manufacturer instructions. Both CcmI and SirG were shown to interact with SirA. Gal11 and LGF2 served as positive control for interaction. Reciprocal assays in which each protein served as either Bait or Target, were performed and similar results were obtained in either case.

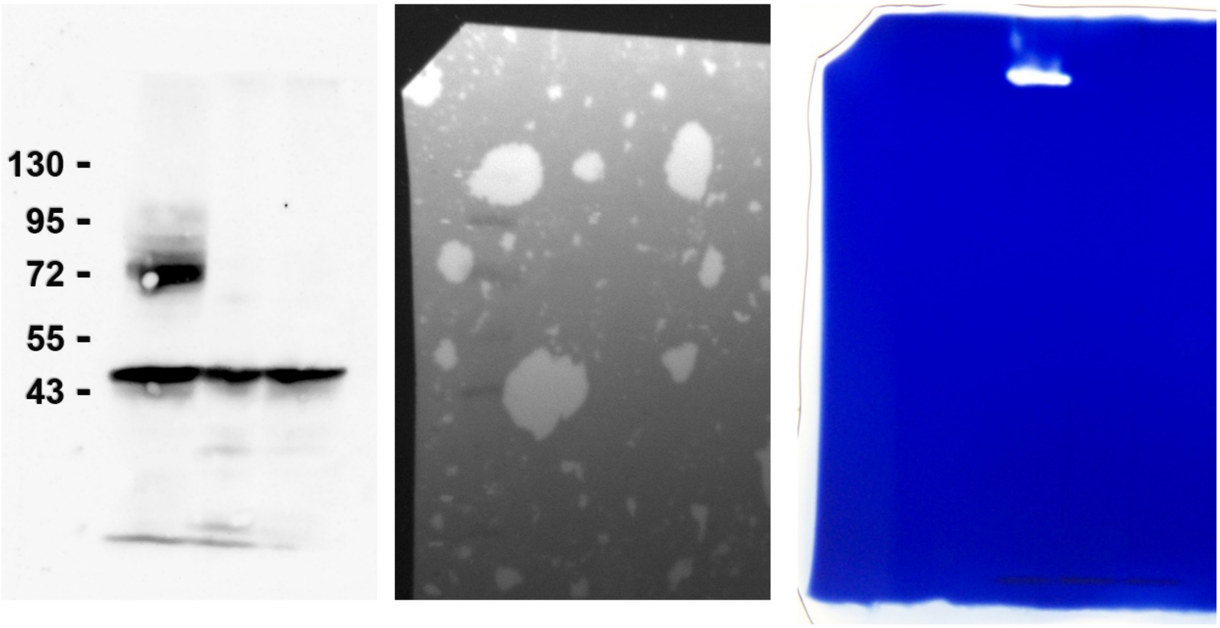

Supplementary Figure S3. Full gel images from bands shown in main text Figure 3. Left panel, western blot with antibodies against SirA peptide. Reactive band at 72 kDa indicated SirA protein. Middle panel, Epiwhite image of blot shown in left panel. Right panel, Native-PAGE sulfite activity gel. Band of clearing indicates sulfite reduction. Lanes for all gels (left to right): Marker, MR1 wildtype, ΔsirA mutant, ΔsirA expressing $SirA_{N589C}$

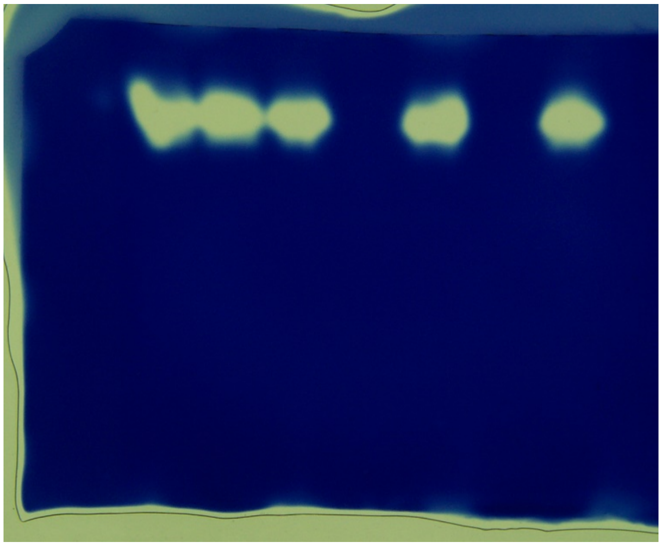

Supplementary Figure S4. Full gel image from bands shown in main text Figure 5. Native-PAGE nitrite activity gel. Band of clearing indicates nitrite reduction. Lanes (left to right): Marker, MR1, ΔsirH, ΔsirEF, ΔnrfA, ΔsirG, ΔccmI, ΔccmI expressing CcmI.

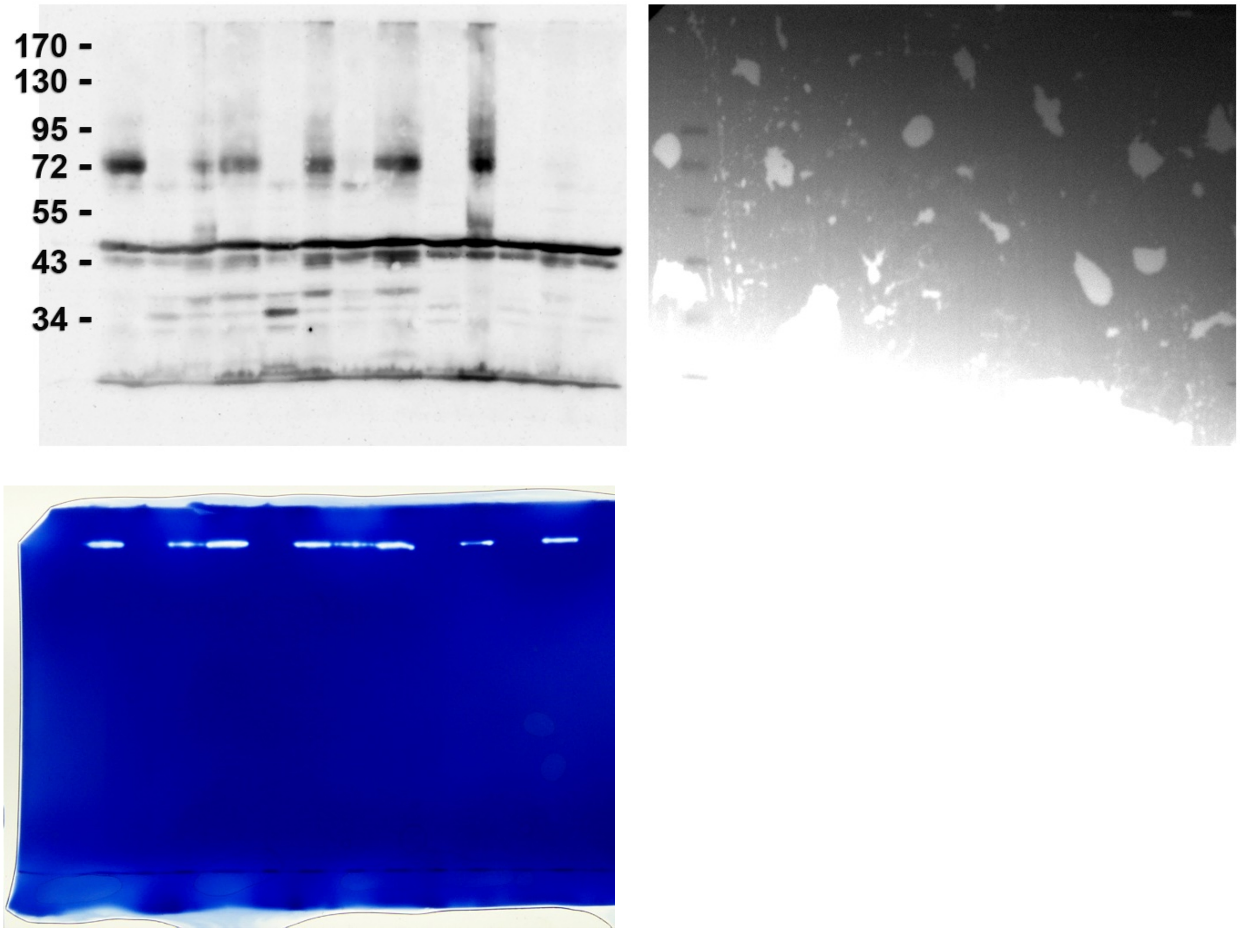

Supplementary Figure S5. Full gel images from bands shown in main text Figure 3. Top left panel, western blot with antibodies against SirA peptide. Reactive band at 72 kDa indicated SirA protein. Top roght panel, Epiwhite image of blot shown in left panel. Lower panel, Native-PAGE sulfite activity gel. Band of clearing indicates sulfite reduction. Lane order for all gels (left to right): Marker, MR1 wildtype, ΔsirA mutant, ΔsirH mutant, ΔsirH complemented with sirH, ΔsirEF mutant, ΔsirEF complemented with sirEF, ΔsirG mutant, ΔsirG complemented with sirG, ΔccmI mutant, ΔccmI complemented with ccmI, ΔsirGΔccmI mutant, ΔsirGΔccmI complemented with ccmI, ΔsirGΔccmI complemented with sirG
